# A Semi-Deterministic Random Walk with Resetting

**DOI:** 10.3390/e23070825

**Published:** 2021-06-28

**Authors:** Javier Villarroel, Miquel Montero, Juan Antonio Vega

**Affiliations:** 1 Instituto Universitario Física y Matemáticas University of Salamanca, Plaza Merced s/n, 37008 Salamanca, Spain; jantovc@gmail.com; 2 Departament de Física de Matèria Condensada, University of Barcelona, Martí i Franquès 1, E-08028 Barcelona, Spain; miquel.montero@ub.edu

**Keywords:** random walk with resetting, escape probabilities, exit times

## Abstract

We consider a discrete-time random walk $\left(x_{t}\right)$ which, at random times, is reset to the starting position and performs a deterministic motion between them. We show that the quantity $\mathrm{Pr}\left(x_{t+1}=n+1|x_{t}=n\right),n\to \infty$ determines if the system is averse, neutral or inclined towards resetting. It also classifies the stationary distribution. Double barrier probabilities, first passage times and the distribution of the escape time from intervals are determined.

## 1. Preliminaries

In a previous paper [1], we introduced the Sisyphus random walk as an infinite Markov chain that moves on the space state N={0,1,2,…,∞} and that, at every step, can either jump one unit rightward or return to the initial state from where it is restarted. The system was named after the king of Ephyra, Sisyphus, who was condemned to lift a heavy stone in an endless cycle.

Here, we generalize the above idea and consider a random walk on the integers ${\left(x_{t}\right)}_{t\in N}$ whose dynamics alternates deterministic linear motion with resets which drive the system to the starting point at the random times ${\left(t_{n}\right)}_{n\geq 1}$. At every clock tick, the position of the random walker is such that $|x_{t}|$ either increases one unit, or returns to the ground state, whereupon the evolution continues. Such resetting occurs through an independent mechanism superimposed onto the original semi-deterministic evolution. Once $\left(x_{t}\right)$ is reset to the origin at $t_{1}$, it begins the evolution anew from scratch which is deterministic between resets.

Using translational invariance, we can suppose that $x_{0}=0$ with no loss of generality. Concretely, starting from $x_{0}=0$, three possibilities exist for the future position $x_{1}$: the system may remain at $x_{1}=0$ provided a reset occurs at t=1; otherwise, it goes one unit to the right with probability ρ or to the left with probability $\bar{\rho }=1-\rho$. In addition, if the system has wandered into the positives so that at a certain time t≥0 is $x_{t}>0$ (respectively $x_{t}<0$) then, at time t+1, it may either be reset to the origin $x_{t+1}=0$ with arbitrary probability or else increase (respectively, decrease) one unit to $x_{t+1}=x_{t}+1$ (respectively $x_{t+1}=x_{t}-1$).

Such apparent simplicity is misleading, as this simple evolution law can exhibit a surprisingly complex and rich behavior. Indeed, at each site, we allow arbitrary probabilities for the random walk to reset to the origin and, additionally, the possibility to move both in the positive and negative integers. The only restriction in this general dynamics is the requirement that ${\left(x_{t}\right)}_{t\in N}$ be a Markov chain. The resulting system is a natural, non-trivial generalization of that of [1], which is recovered when the reset probability is independent of the location and when ρ=1.

In a different setting, such a system may be used as an idealized model of the random dynamics of a “mobile” in a trap, say, who is trying to climb stepwise a ladder or wall given that, at every step, there is a probability of slipping to the bottom, resulting in the need to restart again. Here, the natural question would be the determination of the location probability and expected time to escape the trap.

A related mechanism—Sisyphus cooling— was proposed by Claude Cohen-Tannoudji in certain optical contexts to the effect that an atom may climb up a potential hill, till suddenly it is returned to some ground state where it can restart anew. The hallmark of such systems is the possibility to display “back-to-square-one” behavior, a feature common in real life systems. Indeed, the study of stochastic processes subject to random resets is a problem that has attracted great interest in recent years after the seminal work of Manrubia and Zanette [2] and Evans and Majumadar [3]. Presently, the dynamics of systems with resets is being subjected to intense study, see the recent review [4]. Other mechanisms for random walks that are suddenly refreshed to the starting position are considered in [5,6,7]. Brownian motion with resets is considered in [3,8] while in [9] the propagator of Brownian motion under time-dependent resetting is obtained (see also [10] for further elaboration). In [11], these ideas are applied to the case of a compound Poisson process with positive jumps and constant drift. Further elaboration appears in [12]. Reset mechanisms have also been thoroughly applied to search strategies in mathematical and physical contexts as well as to behavioral ecology, see [10,13,14,15,16,17,18]. Surprisingly, strategies that incorporate reset to pure search are advantageous in certain contexts in ecology and biophysics and molecular dynamics, [19,20,21,22]. A generalization of the Kardar–Parisi–Zhang (KPZ) equation that describes fluctuating interfaces and polymers under resetting is covered in [23]. Dynamical systems with resets have also been used as proxies of the classical integrate-and-fire models of neuron dynamics, see [24,25]. In the context of Lévy flights with resetting, see the interesting papers [26,27]. For other applications, see also the recent papers [28,29,30,31,32,33,34,35].

As commented, the main aim of this paper is to study the main features of the semi-deterministic random walk with resets $(x_{t}),t=0,1,\ldots \infty$. The evolution rules for such a random walk are described in Section 2. We then study the propensity towards resetting of the system. According to this important property, we denote systems as reset averse, neutral or reset-inclined, and characterize them in terms of the transition probabilities and behavior of $\mathrm{Pr}\left(x_{t+1}=n+1|x_{t}=n\right),n\to \infty$. In Section 3, we study the stationary distribution that the system approaches for large time. Section 4 considers first-passage problems and, in particular, two-sided exit probabilities; concretely, given levels a,b∈N, we study the probability that *x* reaches a>0 before having reached −b and distributions of the escape time. First passage times (FPT) also play a key role in statistical decision models, or to devise optimal strategies for seeking information; the rate at which a Brownian particle, under the influence of a metastable potential, escapes from a potential well is also a critical subject in the study of polymers, the so-called Kramers problem [36].

Under the simplest election ρ=1 and $q_{n}:=\mathrm{Pr}\left(x_{t+1}=n+1|x_{t}=n\right)=q_{1}$ constant, we have that the distribution of the FPT to level k≥1 is that of the number of trials required in an unfair coin-toss to obtain *k* consecutive successes, a classical problem in probability. Even with k=2, the distribution of such a problem is not trivial.

## 2. The Model

The evolution rules for the random walk $(x_{t}),t=0,1,\ldots \infty$ are as follows. Let $x_{0}:=0$ be the initial position. We suppose that, if for any t≥0 is $x_{t}=0$, then the random walk satisfies

(1) $$\mathrm{Pr}\left(x_{t+1}=n|x_{t}=0\right)={\bar{q}}_{1}\delta_{n0}+q_{1}\rho \delta_{n1}+q_{1}\bar{\rho }\delta_{n,-1},n\in Z$$

We denote $q_{1}=\mathrm{Pr}\left(x_{t+1}\neq 0|x_{t}=0\right)\in \left(0,1\right)$ the probability that, starting form zero, the system moves away from the origin at the next instant and $\rho :=\mathrm{Pr}\left(x_{t+1}=1|x_{t}=0,x_{t+1}\neq 0\right)\in \left[0,1\right]$ the probability that, if the system abandons the originat time *t*, it goes to position $x_{t+1}=1$. To ease notation, for any value *p*, we set $\bar{p}:=1-p$, ${\bar{q}}_{1}:=1-q_{1}$. Besides $\delta_{nk}$ is the Kronecker delta.

Further, we suppose that the random walk $(x_{t}),t=0,1,\ldots \infty$ is a Markov chain where, if $x_{t}\equiv n\neq 0$, the only allowed transitions are either to site n+sign(n) if no reset occurs, which happens with probability $q_{n+1}$; or else to {0}, when a reset occurs, with probability $1-q_{n+1}$. Here, the sequence $\left(q_{n}\right)$ satisfies that $0\leq q_{n}\leq 1$ for all *n*. It follows that the chain has the transition rules

(2) $$\mathrm{Pr}\left(x_{t+1}=m|x_{t}=n\right)=\left\{\begin{array}{c} q_{n+1},m=n+1\\ 1-q_{n+1},\;m=0 \end{array}\right.,t\geq n>0$$

(3) $$\mathrm{Pr}\left(x_{t+1}=m|x_{t}=n\right)=\left\{\begin{array}{c} q_{|n|+1},m=n-1\\ 1-q_{|n|+1},\;m=0 \end{array}\right.,t\geq -n>0$$

(4) $$\mathrm{Pr}\left(x_{t+1}=m|x_{t}=0\right)=\left\{\begin{array}{c} \rho q_{1},m=1\\ \bar{\rho }q_{1},m=-1\\ 1-q_{1},\;m=0 \end{array}\right.$$

and 0 otherwise. We also suppose that the infinite product with general term $q_{n}$ satisfies

(5) $$\lim_{n\to \infty }\prod_{j=1}^{n}q_{j}=0;\;\mathrm{alternatively}\;\sum_{j=1}^{\infty }\left(1-q_{j}\right)=\infty$$

This mild requirement does not imply that $\lim_{n\to \infty }q_{n}=0$ (see Equation (12) below).

**Figure 1:**
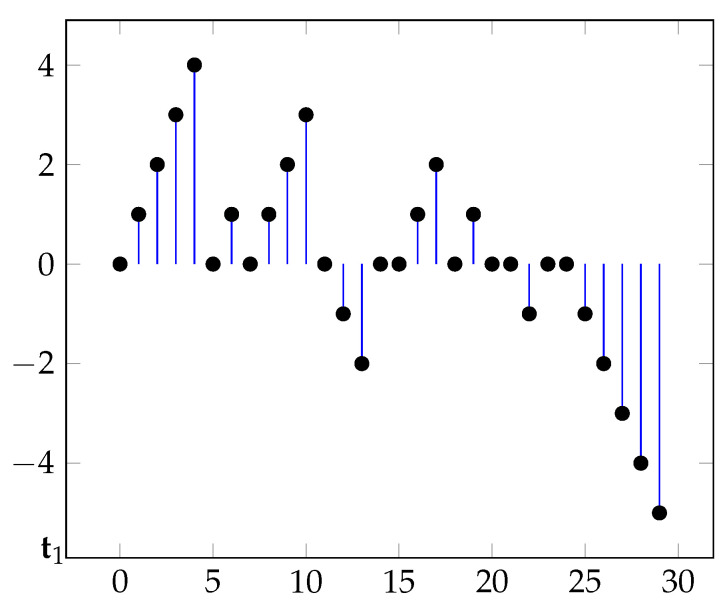
A typical sample paths of the process where $t_{1}=5,t_{2}=7,\ldots$ and $x_{1}=x_{6}=1$.

The model considered in [1] is recovered assuming ρ=1 and that the jump-probability is constant: $q_{1}=q_{2}=\ldots q_{n}=\ldots$. Figure 1 displays a typical sample path.

### 2.1. Reset Times

We denote as $t_{1}$ the random time at which the first reset happens. Here, we consider its distribution probability $p_{n}:=\mathrm{Pr}\left(t_{1}=n\right),n=1,\ldots ,\infty$. Similarly, we denote as $t_{k}$ the random time at which the $k{-}^{\mathrm{th}}$ reset happens. To this end, note that for n=1,2,… the reset takes place at time *n* if in all previous times no reset has occurred—and so $|x_{1}\left|=1,\ldots ,\right|x_{n-1}|=n-1$ and $x_{n}=0$. Thus, we have transitions {0}↦{1}…↦{n−1}↦{0} and the corresponding probability

(6) $$p_{n}:=\mathrm{Pr}\left(t_{1}=n\right)=q_{1}\ldots q_{n-1}{\bar{q}}_{n}$$

where $t_{1}$ is a proper random variable, in view of Equation (5).

The following representation clarifies the meaning and different roles of $\left(p_{n}\right)$ and $\left(q_{n}\right)$

(7) $$p_{n}:=\mathrm{Pr}\left(t_{1}=n\right)=\mathrm{Pr}\left(t_{t}=n\right):=\mathrm{Pr}\left(x_{t+n}=0,x_{t+j}\neq 0,0<j<n|x_{t}=0\right)$$

and

(8) $${\bar{q}}_{n}=\mathrm{Pr}\left(x_{t+n}=0|x_{t}=0,x_{t+j}\neq 0,0<j<n\right)$$

We relate both probabilities. We introduce recursively a sequence $\left(\beta_{n}\right)$ via $\beta_{0}\equiv 1$ and $\beta_{n}:=q_{1}\ldots q_{n},n=1,2,\ldots$. Note then that

$$p_{n}=q_{1}\ldots q_{n-1}{\bar{q}}_{n}=\beta_{n-1}-\beta_{n}$$

This can be inverted as

(9) $$\beta_{n}=p_{n+1}+p_{n+2}+\ldots ={\bar{F}}_{t_{1}}\left(n\right)=1-F_{t_{1}}\left(n\right)$$

where $F_{t_{1}}\equiv F$ is the cumulative distribution function (cdf) of $t_{1}$ and $\bar{F}\left(n\right):=1-F\left(n\right)$. Recalling that $\beta_{n}:=q_{1}\ldots q_{n},$ we finally have that Equation (6) can be inverted as

(10) $$q_{n}=\frac{\bar{F}\left(n\right)}{\bar{F}\left(n-1\right)},n=1,2\ldots$$

### 2.2. Reset Averse and Reset-Inclined Systems

One of the most defining traits in the random walk (2)–(4) is what we call propensity towards resetting, a measure of how likely is that the resetting mechanism is triggered as the time from the last reset increases. We say that a system is inclined towards resetting if such a probability grows as the distance to the origin increases: ${\bar{q}}_{n}<{\bar{q}}_{n+1},$ for all *n*. Intuitively, for a reset-inclined system, the greater the time since the last visit (alternatively, the farthest off) the more anxious to return to the origin becomes the random walk. If this probability decreases (respectively, remains unchanged), we say that the system is reset-averse or reset-neutral. Reset-neutral chains correspond to having $q_{n}=q_{n-1}\equiv q_{1}\in \left(0,1\right)$ for all *n*. This is the choice considered in [1]. In this case

(11) $$\mathrm{Pr}\left(t_{1}=n\right)=q_{1}^{n-1}\left(1-q_{1}\right),\;F\left(n\right)=1-q_{1}^{n}$$

Actually, we are interested in this property for large *n*. We say that a system is ultimately averse, neutral or, respectively, inclined towards resetting if, as the time from the last reset tends to infinity, the reset probability $\left(q_{n}\right)$ satisfies

(12) $$\lim_{n\to \infty }q_{n}:=\lim_{n\to \infty }\mathrm{Pr}\left(x_{t+n}\neq 0|x_{t}=0,x_{t+j}\neq 0,0<j<n\right)=\left\{\begin{array}{c} 0,\;\left(\mathrm{inclined}\right)\;\\ q_{\infty }\in \left(0,1\right)\;\left(\mathrm{neutral}\right)\;\\ 1,\left(\mathrm{averse}\right)\; \end{array}\right.$$

The selection $q_{n}=q_{1}/n$ corresponds to an ultimately reset-inclined system. Here, we have $\lim_{n\to \infty }q_{n}=0$ and

(13) $$p_{n}=\frac{q_{1}^{n-1}}{(n-1)!}-\frac{q_{1}^{n}}{n!},\;\bar{F}\left(n\right)=\frac{q_{1}^{n}}{n!},\;n=1,2\ldots$$

A simple calculation yields $<t_{1}>=e^{q_{1}}\leq e$, which is bounded with respect to the parameter $q_{1}$.

Finally, the choice $q_{n}=n/\left(n+1\right)$ corresponds to a reset-averse system. Here, the chain has power law decay tails:

(14) $$p_{n}=\frac{1}{n(n+1)}\;\mathrm{and}\;\bar{F}\left(n\right)=\frac{1}{n+1}$$

The selections (13) and (14) reflect that the probability to commit an error that sends the walker to square one diminishes (increases) with every step. This may be put down to a capability to learn or, in contrast, to forget or grow tired with the distance to the origin. Equation (14) corresponds to $q_{n}=q_{n-1}\left(1+\frac{1}{n^{2}-1}\right)$—and hence to learning—while, if $q_{n}=q_{n-1}\left(1-\frac{1}{n}\right)$, then Zipf law (13)$:q_{n}=q_{1}/n$ follows.

Equation (14) may also arise due to uncertainty in the relevant parameters. Suppose we accept the basic model (11) to hold but are ignorant of the value of parameter $q_{1}$. Besides, we accept that all values for $q_{1}$ are equally likely; in this situation, the parameter $q_{1}$ should be assumed to have Uniform (0,1) distribution. Bayes theorem implies that the distribution at posteriori of $t_{1}$ must be given by Equation (14):

(15) $$\mathrm{Pr}\left(t_{1}=n\right)=\int_{0}^{1}\mathrm{Pr}\left(t_{1}=n|q_{1}\right)dq_{1}=\int_{0}^{1}dq_{1}q_{1}^{n-1}\left(1-q_{1}\right)=\frac{1}{n(n+1)}$$

We next show that the above behavior is ubiquitous, so the reset propensity is directly related with the tail’s behavior. Indeed, since the sequence $\bar{F}\left(n\right)$ is strictly monotone and $\bar{F}\left(n\right)↓0$ as n→∞, the Stolz–Cesáro theorem gives

(16) $$q_{\infty }:=\lim_{n\to \infty }q_{n}=\lim_{n\to \infty }\frac{\bar{F}\left(n\right)}{\bar{F}\left(n-1\right)}=\lim_{n\to \infty }\frac{p_{n}}{p_{n-1}}$$

Requiring $q_{\infty }\equiv e^{-\lambda }\in \left(0,1\right)$, we obtain that asymptotically $\left(p_{n}\right)$ must grow as

(17) $$p_{n}\approx ce^{-\lambda n},c,\lambda >0,\;n\to \infty$$

which is the paradigmatic example of ultimately neutral systems. Note that such $\left(p_{n}\right)$ has medium tails. By contrast, tails of the form

(18) $$p_{n}\approx ce^{-\lambda n^{\alpha }},\;n\to \infty \;\mathrm{where}\;c>0,\lambda >0,\alpha >0$$

give $q_{\infty }=1$ if 0<α<1 and $q_{\infty }=0$ if α>1. The exponential case α=1, i.e., the geometric distribution, marks the crossover between these cases.

Note that slowly, power-law decaying, sequences such as

(19) $$p_{n}\approx c/n^{\alpha },c>0,\alpha >1,\;n\to \infty$$

also correspond to reset-averse systems ultimately. Thus, heavy tails of the sequence $\left(p_{n}\right)$ correspond to reset-averse systems while the opposite holds with medium and light (super-exponential) tails such as those in Equations (11) and (18). Table 1 summarizes all the possibilities.

More complicated tails can be handled noting the behavior of ultimately averse, neutral or inclined reset systems under sums and products. We use $q_{\infty }:=\vartheta$ to denote that $\lim_{n\to \infty }q_{n}\in \left(0,1\right)$ (thus, $\lim_{n\to \infty }q_{n}=0,\vartheta$ or 1). Hence, with obvious notation, the sums and product rules for $q_{\infty }^{\left(1\right)},q_{\infty }^{\left(2\right)}$ read

0+0=0;0+ϑ=ϑ;0+1=1;ϑ+ϑ=ϑ;ϑ+1=1+1=1;

0·0=0·ϑ=0·1=0;ϑ·ϑ=ϑ·1=ϑ;1·1=1

where the symbol ϑ·ϑ=ϑ is used to mean that if

$$\lim_{n\to \infty }q_{n}^{\left(1\right)}\in \left(0,1\right),\lim_{n\to \infty }q_{n}^{\left(2\right)}\in \left(0,1\right),\;\mathrm{then}\;\lim_{n\to \infty }q_{n}^{\left(1\right)}q_{n}^{\left(2\right)}\in \left(0,1\right)$$

As an example, for 0<c<1, consider the hybrid system

$$p_{n}=\frac{n\bar{\nu }+1}{n(n+1)}\nu^{n-1}=O\left(\frac{e^{-\lambda n}}{n}\right)$$

where $\nu :=e^{-\lambda },\lambda >0$. Here, $p_{n}\equiv p_{n}^{\left(1\right)}p_{n}^{\left(2\right)}$ and tails display mixed exponential and power-law decay. Hence, $q_{\infty }=q_{\infty }^{\left(1\right)}\cdot q_{\infty }^{\left(2\right)}=\vartheta \cdot 1=\vartheta$ correspond to an ultimately neutral system. This is corroborated by an exact evaluation of $q_{n}$. Equation (10) yields that

$$q_{n}=n\nu /\left(n+1\right)\;\mathrm{and}\;\;\lim_{n\to \infty }q_{n}=\nu \in \left(0,1\right)$$

### 2.3. Comments on Markovianness

The fact that the system has a capability for learning (or to forget, see Equations (11), (13) and (14)) suggests the existence of some “memory” in the dynamics, a fact that cast doubt upon the Markovian nature of the model. Actually, there is some hidden memory, although not in $\left(x_{t}\right)$.

This apparent paradox has interesting implications and can be developed further as we now explain. At any time *t*, let $N_{t}$ be the process that counts the number of resets $t_{n}$ “observed” in the time window (0,t]. Thus, $N_{t}=n$ if $t_{n}\leq t<t_{n+1}$. Given that $N_{t}=n,n\leq t$ any additional information may result in information relevant to predict its future: if additionally, say $N_{t-1}=n-1$, we infer that a reset has occurred exactly at *t*, a valuable information. Hence, $\left(N_{t}\right)$ is generically non-Markovian. The only exception is the system (11); here, $N_{t}$ follows a binomial distribution with success parameter ${\bar{q}}_{1}$:

(20) PNt=n=tn·q¯1nq1t−n,n=0,1,…t

By contrast, suppose we know $x_{t}=i,0<i\leq t$. This information amounts to saying that the previous reset occurred at $t_{n}=t-i$ and hence pins down the history of the process after $t_{n}$, viz $x_{t-i+j}=i+j,\forall j=0,1,\ldots i$. By contrast, the history of the process previous to $t_{n}$ remains unknown. Should additional information be provided, it would not help predict the future of *x*, since at $t_{n}$ the process started anew; hence, only the history after $t_{n}$ is relevant, but this is already known. Thus, we arrive at the counter-intuitive fact that $\left(N_{t}\right)$ needs not be Markovian but $\left(x_{t}\right)$ is. Likewise, the vector chain $\left(N_{t},x_{t}\right)$ is Markovian.

## 3. Equilibrium Distribution

Here, we consider the large time distribution of the random walk. Call $x_{\infty }\equiv \lim_{t\to \infty }x_{t}$ the limit of the process and $\pi_{n}:=\mathrm{Pr}\left(x_{\infty }=n\right),n\in Z$ its distribution. When it exists it is also a stationary state, in the sense that if it has initially this distribution then it will not abandon it. $\left(\pi_{n}\right)$ satisfies the system

(21) $$\sum_{n\in Z}g_{nm}\pi_{n}=\pi_{m},\;m\in Z$$

where $\left(g_{nm}\right)$ is the transition probability matrix defined in Equation (3):

(22) $$g_{nm}:=\mathrm{Pr}\left(x_{t+1}=m|x_{t}=n\right)$$

To handle this, we divide the matrix in upper and lower parts, connected only by the column and rows with index 0, i.e.,

(23) $$G=\left(\begin{array}{cc} G_{-} & 0\\ 0 & G_{+} \end{array}\right)$$

where $G_{-}$ is essentially obtained from $G_{+}$ by reflection and $G_{+,nm},n,m=0,\infty$ reads (including the 0− column)

(24) $$G_{+}=\left(\begin{array}{cccccc} {\bar{q}}_{1} & \rho q_{1} & 0 & 0 & 0 & \ldots \\ {\bar{q}}_{2} & 0 & q_{2} & 0 & 0 & \ldots \\ {\bar{q}}_{3} & 0 & 0 & q_{3} & 0 & \ldots \\ \ldots & & \ldots \\ {\bar{q}}_{n} & 0 & \ldots & 0 & 0 & q_{n} \end{array}\right)$$

By insertion, we find

$$\pi_{1}=\rho q_{1}\pi_{0},\;\pi_{-1}=\bar{\rho }q_{1}\pi_{0}$$

along with the recursive system

(25) $$\pi_{n+1}=q_{n+1}\pi_{n},n\geq 1\;\mathrm{and}\;\pi_{n-1}=q_{|n|-1}\pi_{n},n\leq -1$$

Solving recursively, we find

(26) $$\pi_{n}=\rho \pi_{0}q_{1}q_{2}\ldots q_{n}=\pi_{0}\rho \bar{F}\left(n\right)\;\mathrm{and}\;\pi_{-n}=\pi_{0}\bar{\rho }\bar{F}\left(n\right),n\geq 1$$

Normalization gives $1/\pi_{0}=\sum_{n=0}^{\infty }np_{n}\equiv <t_{1}>\equiv \mu$ which requires $<t_{1}>\equiv \mu <\infty$, i.e., $\left(p_{n}\right)$ must decay at least as $p_{n}\approx 1/n^{r},r>2$. In this case, defining $\rho_{n}\equiv \rho 1_{n>0}+\bar{\rho }1_{n<0}+\delta_{n0}$, the stationary distribution is

(27) $$\pi_{n}=\left(\rho_{n}/\mu \right)\bar{F}\left(|n|\right),n=-\infty ,\ldots ,\infty$$

The probability that the random walk has drifted to site n for large times decreases as $\bar{F}\left(n\right)$ does, see Table 1. Hence, for reset-averse systems, $\left(\pi_{n}\right)$ displays heavy tails (which may even decay in a weak, algebraic way), and so there exists a non-negligible probability to find the system away from the origin at large times. This agrees with the “unwillingness” of the system to return to the origin. The opposite holds for reset-inclined systems where this probability will decrease quicker than exponentially. Concretely, for the cases (11) and (13), the stationary distribution is

(28) $$\pi_{n}=\rho_{n}\left(1-q_{1}\right)q_{1}^{\left|n\right|}\;\mathrm{and}\;\pi_{n}=\rho_{n}e^{q_{1}}\frac{q_{1}^{\left|n\right|}}{|n|!},\;n=-\infty ,\ldots 0,\ldots ,\infty$$

Finally, for system (14), there is neither an equilibrium nor a stationary distribution, indicating that the chain spreads out far from the origin and does not settle to an equilibrium.

Writing Equation (21) as $\sum_{n\neq m}\pi_{m}g_{nm}=\sum_{n\neq m}\pi_{n}g_{mn}$, it states that the total probability flux from all states *n* into *m*, n≠m, must equal the flux from state *m* into the rest of states. Hence, $\left(\pi_{n}\right)$ satisfies a global balance equations, viz Equation (21). This does not imply that $\left(\pi_{n}\right)$ is an equilibrium state, only a limit state; for an equilibrium distribution it must satisfy the far stronger “detailed balance” condition: $\pi_{m}g_{nm}\neq \pi_{n}g_{mn}$—which does not hold. This was to be expected since detailed balance guarantees time-reversibility of the dynamics, a trait that the system at hand clearly does not exhibit, as a simple inspection of the trajectories shows.

In the absence of any information, the maximum entropy principle yields that the distribution with maximum entropy should be chosen on the basis that it is the least-informative one. Within the class with fixed finite mean, it is well known that this corresponds to a geometric distribution. It follows from Equation (28) that this implies that the model satisfies Equation (11). We conclude that the reset neutral chain (11) satisfying $q_{n}=q_{1}$, for all *n*, and arbitrary ρ has the remarkable properties of being the only selection that corresponds to a truly Markovian situation (i.e., for both $\left(x_{t}\right)$ and the arrival process $\left(N_{t}\right)$) and the one that gives the maximum entropy for fixed mean.

## 4. Escape Probabilities

In a classical study, W. Feller [37] showed that most recurrent properties of general diffusion processes can be codified in terms of two of the functions that define escape probabilities from an interval (c,d),c<d. Given that the process has started from a general $x_{0},c<x_{0}<d$, Feller considers the “scale and speed functions”, defined as

(29) $$s\left(x_{0}\right)=\mathrm{Pr}\left(\tau_{d}>\tau_{c}\right)\;\mathrm{and}\;m\left(x_{0}\right)\equiv <\min\left\{\tau_{c},\tau_{d}\right\}>$$

and shows that they solve certain differential equations (see [37] for an overview). Here, for any a∈R, we introduce the “hitting time” $\tau^{a}=\inf\left\{t>0:x_{t}=a\right\}$ which represents the lapse of time necessary to travel from the starting value to *a*.

We perform a similar study here and determine, for given levels a,b∈N,−b<0<a, the probability that the random walk $\left(x_{t}\right)$ reaches *a* before having reached −b. Note that by translational invariance the case when (x) starts from general $x_{0}$ is immediately reduced to that with $x_{0}=0$.

We start noting that when resets are switched off the only source of randomness lies in the first displacement of the random walk away from x=0; hence, $x_{n}=n$ for all *n* if $x_{1}=1$. In this case, $\tau_{a,b}$—the minimum time to hit either a>0 or −b<0—is a binary random variable that takes values *a* and *b* with probabilities ρ and $\bar{\rho }$. Besides, ${\mathrm{Pr}}_{0}\left(\tau_{a}<\tau_{b}\right)=\rho$.

Obviously, $\tau_{a,b}$ will increase when a reset mechanism is introduced; it is, however, tempting to think that resets do not affect the escape probabilities, namely ${\mathrm{Pr}}_{0}\left(\tau_{a}<\tau_{b}\right)=\rho$ still holds. However this is not correct! To dispel such a misinterpretation, note that resets introduce a bias which favors the closest barrier against the farthest one. This is similar to the classical waiting time paradox where cycles with very large inter-reset times have a greater probability than smaller ones. Intuitively, if restarts occur very often, the possibility to reach the farthest barrier diminishes. We now determine this probability.

A very simple argument goes as follows. Consider the probability $\ell_{a}$ that the random walk (x) reaches a>0 before having reached −b when we know that $\left(x_{t}\right)$ hits *a* or −b in the given cycle. The event that escape occurs at a given cycle, say, the first, is

(30) $$E:=\left\{x_{1}>0,t_{1}>a\right\}\cup \left\{x_{1}<0,t_{1}>b\right\}\equiv E_{1}\cup E_{2}$$

with probability

(31) $$\kappa \equiv \mathrm{Pr}\left(E\right)=\rho \bar{F}\left(a\right)+\bar{\rho }\bar{F}\left(b\right)$$

Hence, the probability that escape occurs via the upper barrier can be evaluated as the probability of $E_{1}$ conditional on the event *E* having happened:

(32) $$\ell_{a}=\mathrm{Pr}\left(\;\mathrm{escape}\;\mathrm{via}\;a|E\right)=\mathrm{Pr}\left(E_{1}|E_{1}\cup E_{2}\right)=\frac{\rho \bar{F}\left(a\right)}{\kappa }$$

The reasoning when escape occurs at a general given cycle is a bit more involved but does not change the result.

Denote $\ell_{a}^{0}\equiv \rho$ the corresponding probability when no resets are introduced. Then,

(33) $$\ell_{a}\geq \ell_{a}^{0}\Leftrightarrow \bar{F}\left(b\right)\leq \bar{F}\left(a\right)\Leftrightarrow b\geq a$$

which means that resets increase the probability to hit first the closest barrier, as expected. Further, when a=b Equation (32) yields $\ell_{a}=\ell_{a}^{0}$.

We thus have for the neutral chain (11), the reset-averse chain (13) and the reset-inclined chain (14), respectively

(34) $$\ell_{a}=\left\{\begin{array}{c} \rho {\left(\rho +\bar{\rho }q_{1}^{b-a}\right)}^{-1}\\ \rho b!{\left(b!\rho +\bar{\rho }a!q_{1}^{b-a}\right)}^{-1}\\ {\left(1+\frac{\bar{\rho }\left(a+1\right)}{\rho (b+1)}\right)}^{-1} \end{array}\right.=\left\{\begin{array}{c} \rho {\left(\rho +\bar{\rho }q_{1}^{d}\right)}^{-1}\\ \rho {\left(\rho +\bar{\rho }q_{1}^{d}a!/\left(a+d\right)!\right)}^{-1}\\ {\left(1+\frac{\bar{\rho }\left(a+1\right)}{\rho (a+d+1)}\right)}^{-1} \end{array}\right.$$

In the second equality, we introduce d:=b−a, which measures the departure from symmetry of the problem and suppose b≥a for ease of notation.

## 5. Escape Times

### 5.1. Symmetry Properties of First Passage Times

Denote for a moment as $\tau_{a,b}^{\rho }$ the FPT to either *a* or −b when $\mathrm{Pr}(x_{t+1}=1|x_{t}=0,x_{t+1}\neq 0)=\rho$. This quantity $\tau_{a,b}$ has a nice interpretation. Suppose the model (11) holds. Say a success has been scored every time a reset does not happen. Then, $\tau_{n,n}^{1}=\tau_{n}^{1}$ is the time that takes to obtain n≥1 successes in a row provided the probability of individual success is $q_{1}$, a classical problem in probability. Obviously, if n=1 then $\tau_{1}^{1}$—the first time to reach level 1—must have a geometric distribution with parameter $q_{1}$. However, even with n=2, this problem has no easy solution, not even for the mean times.

We note the interesting relation between the asymmetric and symmetric cases.

1. If $l_{a}$ is defined in Equation (32) and $l_{a}+l_{b}=1$ and EX≡<X> indicates the expected value of the random variable *X*, we have
  (35) $$E\tau_{a,b}^{\rho }=l_{a}E\tau_{a,a}^{\rho }+l_{b}E\tau_{b,b}^{\rho }\;$$
2. $\tau_{a,a}^{\rho }$ is independent of ρ. Besides, the distributions in the symmetric case and one-sided case are equal, namely, for any *b*
  (36) $$\tau_{a,a}^{\rho }=\tau_{a,b}^{1}=\tau_{a,\infty }^{1}\equiv \tau_{a}^{1}\;;\tau_{a,\infty }^{\rho }=\tau_{a}^{\rho }\;$$
  Indeed, when the interval is symmetric the escape time will not be influenced by whether resets favor upward or downward flights; hence, Equation (36) must hold. For the sake of comparison, we see that Equation (39) overestimates the time it takes to reach the boundaries.

A first approximation is given by $<\tau_{a,b}>\approx <N>\times <t_{1}>$ where N is the number of resets until escape. To warm up, we consider first the distribution of N.

By independence of cycles, N has a geometric distribution with exit parameter κ:=Pr(E) where *E*, κ are defined in Equations (30), (31). Thus, we have N∼ Geom (κ):

(37) $$\mathrm{Pr}\left(N=n\right)=\kappa {\left(1-\kappa \right)}^{n-1},n=1,2\ldots \;\mathrm{and}\;$$

(38) $$<N>=\left(1/\kappa \right),\;<\tau_{a,b}>\approx <N>\times <t_{1}>=\frac{\sum_{n=1}^{\infty }np_{n}}{\rho \bar{F}\left(a\right)+\bar{\rho }\bar{F}\left(b\right)}$$

In particular, for the symmetric case a=b,

(39) $$<\tau_{a,b}>\approx \left(\sum_{n=1}^{\infty }np_{n}\right)/\left(\sum_{n=a+1}^{\infty }p_{n}\right)\geq a+\left(\sum_{n=1}^{a}np_{n}\right)/\left(\sum_{n=a+1}^{\infty }p_{n}\right)$$

Clearly, this approximation is only reasonable when the system needs a large number of resets prior to exit the interval, i.e., κ≈0.

### 5.2. Mean Exit Time

To study the exact time to hit *a* or *b*, we note that depending of what happens at the the first reset $t_{1}$ there are five excluding and exhausting possibilities. These scenarios are:

- (S1) $x_{1}>0$ and $t_{1}>a$;
- (S2) $x_{1}<0$ and $t_{1}>b$;
- (S3) $x_{1}>0$ and $t_{1}\leq a$;
- (S4) corresponds to having $x_{1}<0$ and $t_{1}\leq b$;
- (S5) corresponds to $x_{1}=0$.

Under scenario (S1), $\left(x_{t}\right)$ hits *a* before it hits *b* with $\tau_{a,b}=a$. Under scenario (S2), $\left(x_{t}\right)$ hits *b* before *a* and $\tau_{a,b}=b$. Scenarios (S3) to (S5) refresh $\left(x_{t}\right)$ to the origin so the “race” starts again from scratch; hence $\tau_{a,b}=t_{1}+\tau_{a,b}^{\prime }$, where $\tau_{a,b}^{\prime }$ is the time that remains until exit once the new cycle starts. This implies that

(40) $$\tau_{a,b}=\left\{\begin{array}{c} a\;\mathrm{if}\;x_{1}>0,t_{1}>a\\ b\;\mathrm{if}\;x_{1}<0,t_{1}>b\\ t_{1}+\tau_{a,b}^{\prime }\;\mathrm{if}\;2\leq t_{1}\leq a,x_{1}>0\;\mathrm{or}\;2\leq t_{1}\leq b,x_{1}<0\;\mathrm{or}\;t_{1}=1 \end{array}\right.$$

$$\;\mathrm{and}\;E\tau_{a,b}=a\rho \bar{F}\left(a\right)+b\bar{\rho }\bar{F}\left(b\right)+$$

$$\rho E\left(t_{1}1_{t_{1}\leq a}\right)+\bar{\rho }E\left(t_{1}1_{t_{1}\leq b}\right)+\left(\bar{\rho }F\left(b\right)+\rho F\left(a\right)\right)E\tau_{a,b}^{\prime }$$

Here, $1_{A}=1$ if the event *A* holds and is 0 otherwise. Thus, we finally obtain

(41) $$E\tau_{a,b}=\frac{\rho \left(a\bar{F}\left(a\right)+E\left(t_{1}1_{t_{1}\leq a}\right)\right)+\bar{\rho }\left(b\bar{F}\left(b\right)+E(\left(t_{1}1_{t_{1}\leq b}\right)\right)}{\bar{\rho }\bar{F}\left(b\right)+\rho \bar{F}\left(a\right)}$$

If b→∞, then $b\bar{F}\left(b\right)\to 0$ and we recover the mean hitting time to level *a* as

(42) $$E\tau_{a}=a+\frac{1}{\rho \bar{F}\left(a\right)}\left(E\left(t_{1}\right)-\rho E\left(t_{1}1_{t_{1}>a}\right)\right)$$

Particularly interesting is the symmetric case a=b. Here,

(43) $$E\tau_{a,a}^{\rho }=E\tau_{a}^{1}=a+\frac{E(t_{1}1_{t_{1}\leq a})}{\bar{F}\left(a\right)}=a+\left(\sum_{n=1}^{a}np_{n}\right)/\left(\sum_{n=a+1}^{\infty }p_{n}\right)$$

Note how this implies equation (35).

### 5.3. Distribution of the Exit Time

Finally, we consider the distribution of $\tau_{a,b}$. We evaluate its generating function

(44) $$G_{}\left(z\right)=\sum_{n=1}^{\infty }z^{n}\mathrm{Pr}\left(\tau_{a,b}=n\right)$$

by using Equation (40). Here, z∈C,|z|≤1. Recall that

(45) $$\tau_{a,b}=a1_{x_{1}>0,t_{1}>a}+b1_{x_{1}<0,t_{1}>b}+\left(t_{1}+\tau_{a,b}^{\prime }\right)$$

$$1_{2\leq t_{1}\leq a,x_{1}>0}+1_{2\leq t_{1}\leq b,x_{1}<0}+1_{t_{1}=1}$$

Note also that

$$E\left(z^{t_{1}+\tau_{a,b}^{\prime }}1_{2\leq t_{1}\leq a,x_{1}>0}\right)=E\left(z^{t_{1}}1_{2\leq t_{1}\leq a,x_{1}>0}\right)E\left(z^{\tau_{a,b}}\right)=\rho {\hat{p}}^{a}\left(z\right)G_{\tau_{a,b}}\left(z\right)$$

where we define the truncated generating function ${\hat{p}}^{a}\left(z\right)=\sum_{k=1}^{a}z^{k}p_{k}$.

It follows from Equation (40) that $G_{\tau_{a,b}}\left(z\right)$ is the sum of the following terms

(46) $$G_{\tau_{a,b}}\left(z\right)=E_{1}+E_{2}G_{\tau_{a,b}^{\prime }}\left(z\right)$$

where

$$E_{1}:=z^{a}\mathrm{Pr}\left(x_{1}>0,t_{1}>a\right)+z^{b}\mathrm{Pr}\left(x_{1}<0,t_{1}>b\right)=z^{a}\rho \bar{F}\left(a\right)+z^{b}\bar{\rho }\bar{F}\left(b\right),$$

$$E_{2}:=E(z^{t_{1}}1_{t_{1}\leq a,x_{1}>0})+E\left(z^{t_{1}}1_{t_{1}\leq b,x_{1}<0}\right)+E\left(1_{t_{1}=1}\right)$$

Thus finally, in Laplace space, the generating functions read

(47) $$G_{\tau_{a,b}}\left(z\right)=\frac{z^{a}\rho \bar{F}\left(a\right)+z^{b}\bar{\rho }\bar{F}\left(b\right)}{1-\rho {\hat{p}}^{a}\left(z\right)-\bar{\rho }{\hat{p}}^{b}\left(z\right)}$$

Hence, the mass function of $\tau_{a,b}$ is

(48) $$P\left(\tau_{a,b}=n\right)=\frac{\bar{F}\left(a\right)}{2\pi i}\oint dz\frac{G_{\tau_{a,b}}\left(z\right)}{z^{n+1}},n\geq 1$$

If either b=a (symmetric case) or ρ=1,b=1 (one-sided case), it simplifies to

(49) $$G_{\tau_{a,a}}\left(z\right)=\frac{z^{a}\bar{F}\left(a\right)}{1-{\hat{p}}^{a}\left(z\right)}$$

(50) $$P\left(\tau_{a,a}=n\right)=\frac{\bar{F}\left(a\right)}{2\pi i}\oint dz\frac{dz}{z^{n+1-a}\left(1-{\hat{p}}^{a}\left(z\right)\right)},n\geq a$$

The FPT to *a* is recovered letting b→∞; then, ${\hat{p}}^{b}\left(z\right)\to \hat{p}\left(z\right):=\sum_{n=1}^{\infty }z^{n}p_{n}$ and

(51) $$G_{\tau_{a}}\left(z\right)=\frac{z^{a}\rho \bar{F}\left(a\right)}{1-\rho {\hat{p}}^{a}\left(z\right)-\bar{\rho }\hat{p}\left(z\right)}$$

### 5.4. FPT under the Model *(11)*

If Equation (11) holds, the distribution of $\tau_{a,a}$ simplifies. The generating function and distribution of the exit time read ${\hat{p}}^{a}\left(z\right)={\bar{q}}_{1}z\left(1-{\left(zq_{1}\right)}^{a}\right)/\left(1-zq_{1}\right)$ and

(52) $$G_{\tau_{a,a}}\left(z\right)=\frac{{\left(q_{1}z\right)}^{a}\left(1-q_{1}z\right)}{1-z+q_{1}^{a}{\bar{q}}_{1}z^{a+1}}$$

Hence, when a=1 we recover $G_{\tau_{1,1}}\left(z\right)=q_{1}z/\left(1-{\bar{q}}_{1}z\right)$ corresponding to a geometric distribution. Note

(53) $$\mathrm{Pr}\left(t_{1}=n\right)=q_{1}^{n-1}\left(1-q_{1}\right),\;\mathrm{Pr}\left(\tau_{1,1}=n\right)={\bar{q}}_{1}^{n-1}q_{1}$$

For a=2, we have

(54) $$G_{\tau_{2,2}}\left(z\right)=\frac{{\left(q_{1}z\right)}^{2}}{1-{\bar{q}}_{1}z-q_{1}{\bar{q}}_{1}z^{2}}$$

If $s_{\pm }:={\bar{q}}_{1}\pm \sqrt{{\bar{q}}_{1}^{2}+4q_{1}{\bar{q}}_{1}}$, this can be inverted as

(55) P(τ2,2=n)=q12∑j=0n−2n−2−jjq1jq¯1n−2−j=q12s+n−1−s−n−12n−2(s+−s−)

Hence, summing an arithmetic-geometric series, we find if $\ell =1/q_{1}$

(56) $$E\tau_{a,a}=a+\frac{1}{q_{1}^{a}{\bar{q}}_{1}^{2}}\left(q_{1}(1-q_{1}^{a+1}-{\bar{q}}_{1}\left(a+1\right)q_{1}^{a+1}\right)=\frac{\ell^{a}-1}{\ell -1}$$

Let $\xi_{a}$ denote the number of trials until the first consecutive *a* successes occur in a sequence of Bernoulli trials (or unfair coin-tosses) with probability of individual success $q_{1}$. This problem does not have a simple answer except when a=1. Clearly, $\xi_{1}∼\mathrm{Geom}\left(q_{1}\right)$.

To handle the case a≥2, we note that the distribution of $\xi_{a}$ is that of the FPT to level *a* provided ρ=1; it is recovered letting b→∞ (see Equation (36) ) and using Equation (52) as

(57) $$\xi_{a}=\tau_{a,\infty }^{1}=\tau_{a}^{1}=\tau_{a,a}^{\rho }\;\mathrm{and}\;G_{\xi }\left(z\right)=\frac{{\left(q_{1}z\right)}^{a}\left(1-q_{1}z\right)}{1-z+q_{1}^{a}{\bar{q}}_{1}z^{a+1}}$$

The mean number of trials until the first consecutive *a* successes is

$$a+\frac{E(t_{1}1_{t_{1}\leq a})}{\bar{F}\left(a\right)}$$

## 6. Discussion

We considered a discrete-time random walk $\left(x_{t}\right)$ which at random times is reset to the starting position and performs a deterministic motion between them. We discussed how to interpret the property that the system is averse, neutral or inclined towards resetting. We showed that such a behavior is critical for the existence and properties of the stationary distribution. We obtained double barrier probabilities, first passage times and the distribution of the escape time from intervals. We pointed out that the distribution of the FPT to level k≥1 solves a classical problem in probability, namely that of the number of trials required in an unfair coin-toss or Bernoulli trial to obtain *k* successes in a row.

## Figures and Tables

**Table 1:** The table summarizes the propensity to resetting in terms of the decay of $p_{n}:=\mathrm{Pr}\left(t_{1}=n\right)$ and the equilibrium distribution. In all cases λ>0.

$p_{n\to \infty }$	$F_{t_{1}}\left(n\right)$	$q_{n\to \infty }$	Propensity	Tails	$Et_{1}$	$\pi_{n\to \infty }$
$O(e^{-\lambda n^{\alpha }}),\alpha >1$	$O(e^{-\lambda n^{\alpha }})$	0	inclined	Super-exp.	<*∞*	$O(e^{-\lambda n^{\alpha }})$
$O({\left(\frac{e^{-\lambda }}{n}\right)}^{n},$	$O({\left(\frac{e^{-\lambda }}{n}\right)}^{n},$	0	inclined	Super-exp.	<*∞*	$O({\left(\frac{e^{-\lambda }}{n}\right)}^{n}$
$O(e^{-\lambda n})$	$O(e^{-\lambda n})$	∈(0,1)	neutral	exp.	<*∞*	$O(e^{-\lambda n})$
$O(e^{-\lambda n^{\alpha }}),0<\alpha <1$	$O(e^{-\lambda n^{\alpha }}),0<\alpha <1$	1	averse	Sub-exp.	<*∞*	$O(e^{-\lambda n^{\alpha }})$
$O(1/n^{\alpha }),\;\alpha >2$	$O(1/n^{\alpha -1}),\;\alpha >2$	1	averse	Power-law	<*∞*	$O(n^{1-\alpha })$
$O(1/n^{\alpha }),\;1<\alpha \leq 2$	$O(1/n^{\alpha -1}),\;1<\alpha \leq 2$	1	averse	Power-law	=*∞*	—-

## Data Availability

No new data were created or analyzed in this study. Data sharing is not applicable to this article.
